# A transcription factor is the target of propranolol treatment in infantile hemangioma

**DOI:** 10.1172/JCI156863

**Published:** 2022-02-01

**Authors:** Sandra Schrenk, Elisa Boscolo

**Affiliations:** 1 Division of Experimental Hematology and Cancer Biology, Cincinnati Children’s Hospital Medical Center, Cincinnati, Ohio, USA.; 2 Department of Pediatrics, University of Cincinnati College of Medicine, Cincinnati, Ohio, USA.

## Abstract

Propranolol is a nonselective β-adrenergic receptor (AR) blocker that has been the first-line therapy for problematic infantile hemangioma (IH), the most frequent childhood vascular tumor. Although IHs are benign and eventually regress spontaneously, at least 15% of patients require treatment. Despite the extensive use of propranolol for IH treatment, its mode of action remains unclear. In this issue of the *JCI*, Seebauer et al. investigated the cellular and molecular consequences of propranolol treatment on IH vascular tumor formation in a murine model of IH. The efficacy of propranolol was independent of its β-AR blocker activity and was attributable to the direct targeting of the transcription factor SOX18, which, in turn, reduced hemangioma blood vessel formation. We believe these results will guide clinical translation for the use of more efficient and safer therapies for IH and possibly for other vascular anomalies in which SOX18 plays a role.

## Infantile hemangioma is a vascular tumor

Infantile hemangioma (IH) is the most frequent tumor of infancy, affecting up to 5% of newborns. IH is more prevalent in White children and premature newborns and is three times more common in female than male infants. IH lesions are disfiguring, and approximately 10% to 15% of cases can be serious or associated with life-threatening complications including bleeding, airway obstruction, respiratory distress, congenital heart failure, and risk to organ development and function (1). IH tumors appear shortly after birth and follow a distinctive life cycle characterized by rapid growth during the first 9 to 12 months followed by a slow and spontaneous regression. During the proliferative phase, immature hyperproliferative endothelial cells (ECs) are arranged into densely packed and highly disorganized blood vessels. The IH regression phase, which is referred to as involution, can last several years and is characterized by the maturation and subsequent apoptosis of these blood vessels followed by infiltration of fibroblasts and adipocytes. It is important to note that substantial structural abnormalities and disfigurement often persist in children after the tumor involutes. Prior to the discovery of propranolol’s efficacy in the treatment of IH, approaches for managing complicated IHs included systemic or intralesional corticosteroids, chemotherapeutic agents such as vincristine, laser therapy, and surgical resection (1).

To date, the genetic determinants of IH are still unknown. Therefore, to improve the understanding of the cellular and molecular mechanisms underlying IH formation and growth, investigations have mostly focused on the isolation and characterization of the cellular components of proliferative tumors. Several studies led by Joyce Bischoff provided evidence that IH building blocks are hemangioma progenitor/stem cells (HemSCs), ECs (HemECs), and pericytes (HemPericytes).

Both vasculogenesis and angiogenesis have been proposed as mechanisms that contribute to neovascularization in IH. HemSCs have the capability to differentiate into ECs and pericytes, suggesting that they can form blood vessels de novo via vasculogenesis. Subsequent sprouting of preformed vessels, including the proliferation of ECs and recruitment of pericytes — via angiogenesis, is thought to further contribute to IH growth during the proliferative phase (2).

## The use of propranolol for vascular diseases

The invention of propranolol in the 1960s revolutionized the treatment of cardiovascular diseases. For this work, James Black was awarded the Nobel Prize in Physiology or Medicine in 1988 (3). Propranolol is a nonselective antagonist that binds with high affinity to both β1- and β2-adrenergic receptor (AR) subtypes, which belong to the superfamily of GPCRs. Propranolol is a chiral drug, meaning it is marketed as a racemate, which is composed of two enantiomers, R(+) and S(–). Most of the β-AR blocking activity resides in the S(–) enantiomer, while the targets of the R(+) enantiomer have not yet been determined (4).

The efficacy of propranolol in IH was discovered serendipitously in 2008, when it was administered to a patient with IH to treat a severe complication that consisted of obstructive hypertrophic cardiomyopathy (5). Results from a subsequent randomized trial showed that 60% of patients with IH responded to treatment, with tumor resolution within six months (6).

The FDA approved the use of propranolol for IH in 2014. Despite its efficacy in treating IH, several known adverse effects have been reported including hypotension, bradycardia, hypoglycemia, and hypoglycemia-induced seizure (6). Thus, it is important to understand the biological mechanisms of action of this medication so that the most effective therapy is given, while minimizing potential adverse effects.

Several mechanisms of action have been proposed for the efficacy of propranolol in IH, including EC apoptosis and inhibition of angiogenesis by modulating vascular endothelial growth factors (7, 8). However, most of these in vitro studies were not reproduced in vivo, and the drug concentrations used were much higher than the plasma levels reported in patients with IH. Hence, the precise mechanism of action of propranolol remained largely unknown.

In this issue of the *JCI*, Seebauer et al. addressed this important question in the field of IH (9). Propranolol and the R(+) enantiomers of both propranolol and atenolol inhibited the differentiation of HemSCs into ECs, thereby preventing vasculogenesis in a well-established murine model of IH in which patient-derived HemSCs were injected subcutaneously into immunocompromised mice (10). There was no difference in the efficacy of the three different treatments, and the response to both R(+) drugs was dose dependent (9). This result suggests that both propranolol and atenolol exert their efficacy through the R(+) enantiomers.

Similar to propranolol, atenolol is a chiral drug composed of S(–) and R(+) enantiomers but is selective for the β1-AR (11). A study by Lee et al. showed that IH cells express very low levels of the β1-AR (12), which would exclude a role for β-AR blockade in IH and thus support the findings by Seebauer et al. that R(+) enantiomers prevent IH vasculogenesis (9).

Propranolol is also being evaluated in a clinical trial for the treatment of another vascular anomaly, cerebral cavernous malformation (CCM) (13). Preclinical studies in CCM murine and zebrafish models demonstrated the efficacy of propranolol in reducing vascular lesion burden and size. In these models, efficacy was associated with the S(–) enantiomer and β1-AR antagonism (14); however, a role for the β-AR–independent activity of propranolol was not directly tested, and we speculate that it cannot be fully excluded.

## SOX18 as a therapeutic target in IH

SOX18 is a transcription factor that acts as a master regulator of vascular development. Pioneering work led by Mathias Francois and other investigators determined that SOX18 is expressed during early vasculogenesis, when it acts as a molecular switch to initiate EC specification (15, 16) as well as lymphangiogenesis (17). Furthermore, SOX18 is an active player during tumor vascularization, and its inhibition can prevent tumor angiogenesis (18).

Transcription factors are proteins that bind to specific DNA sequences and can activate or repress the transcription of a gene. Generally, transcription factors function in a multisubunit protein complex. Mechanistically, SOX18 can regulate endothelial gene transcription by dimerization, which involves protein-protein interactions: two SOX18 proteins can interact with each other to form a homodimer, or a single SOX18 protein can form a heterodimer with other transcription factors. Dimerization of SOX18 with other key endothelial transcription factors refines the selective binding to the DNA to activate the transcription of endothelium-specific genes such as Notch1 (19), VCAM1 (20), Claudin5 (21), and Prox1 (17). Importantly, the Notch signaling regulator recombination signal binding protein for immunoglobulin κ J region (RBPJ) was identified as a SOX18 protein partner (22).

SOX18 dominant-negative gene mutations were discovered in patients affected by a rare condition called hypotrichosis-lymphedema-telangiectasia and renal syndrome (HLTSR). This disease is characterized by severe vascular defects including hemorrhagic blood vessels and lymphedema, defective hair follicle development, and renal defects (23). As for IH, the positive outcome of propranolol treatment in HLTRS was a circumstantial discovery. In 2016, a patient with an HLTRS-causing SOX18 mutation was surprisingly reported to display only mild disease symptoms. Retrospectively, it was revealed that the patient had been treated long term with propranolol for thoracic artery dilation and hypertension. These findings raised the possibility that propranolol may act in a SOX18-dependent fashion (24).

Importantly, these results raised the question of whether R(+) propranolol has a similar mode of action in the treatment of IH. In this issue of the *JCI*, Seebauer et al. (9) demonstrated by innovative real-time, single-molecule tracking imaging that R(+) propranolol can interfere with the binding dynamics of SOX18 to chromatin in live cells. Further mechanistic studies confirmed that R(+) enantiomers of propranolol and atenolol can disrupt SOX18:RBPJ heterodimer and SOX18 homodimer formation.

**Figure 1:**
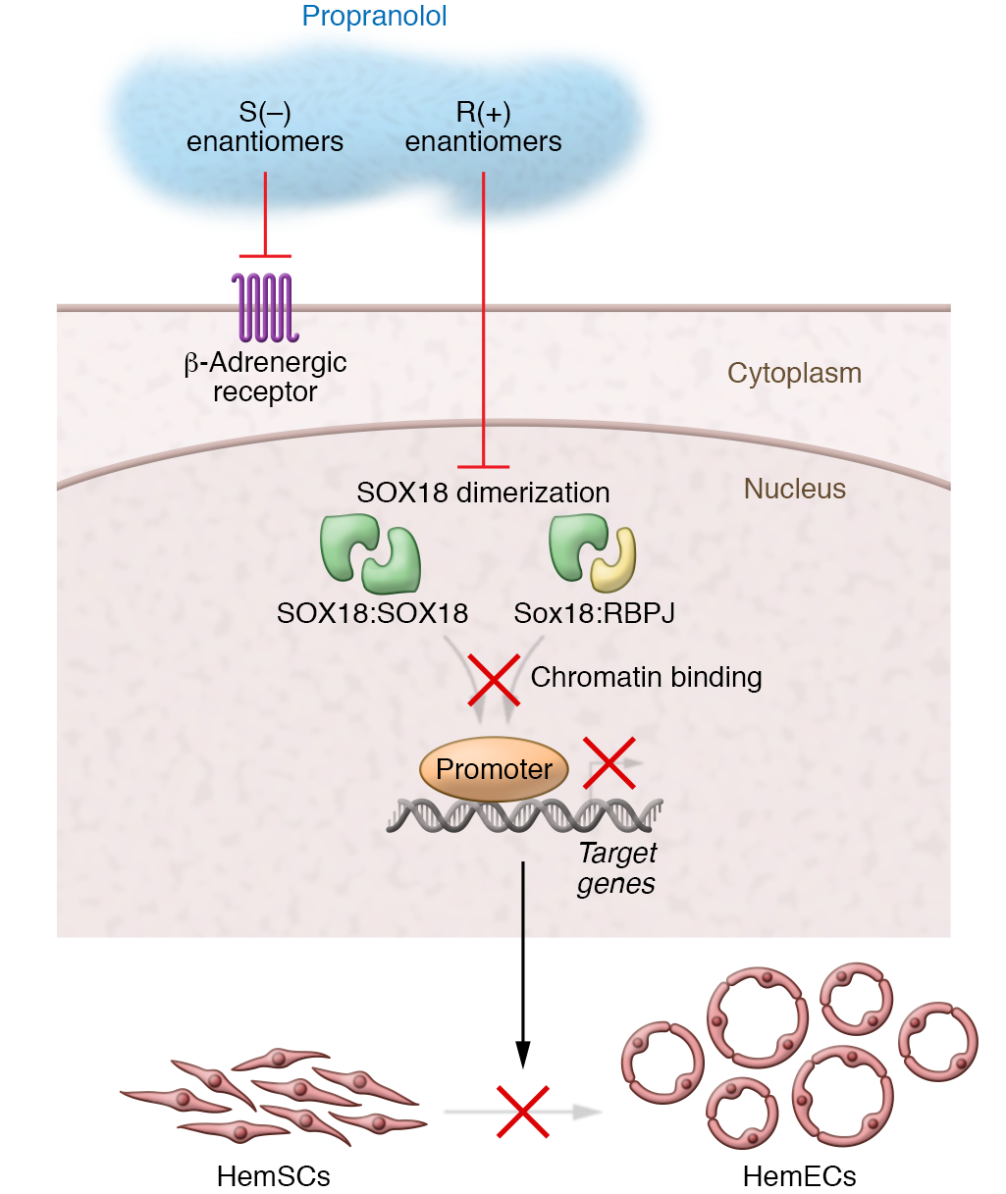
Mechanism of the antiangiogenic effect of propranolol in IH. Propranolol is a mixture of S(–) and R(+) enantiomers. The β-AR–inhibiting function is attributed to the S(–) enantiomer, whereas the R(+) enantiomer mostly lacks β-AR blocker activity. The R(+) propranolol enantiomer inhibits SOX18 homodimer (SOX18:SOX18) and SOX18 heterodimer formation with the RBPJ (SOX18:RBPJ) and impedes chromatin binding. These interferences repress the transcription of SOX18 target genes. Functionally, R(+) propranolol inhibits the differentiation of infantile HemSCs into HemECs, resulting in impaired vasculogenesis in a patient-derived murine model of IH (9).

Consequently, these interferences were shown by the authors to repress the expression of SOX18 direct target genes including Notch1 and VCAM1. Supporting these discoveries, a pharmacological inhibitor of SOX18 called Sm4 (25) was also able to recapitulate the antiangiogenic, preventative effects of the R(+) enantiomers of propranolol and atenolol in the IH murine model (Figure 1).

## Conclusions and future directions

The study by Seebauer et al. provides a major advance in our knowledge of SOX18 as a β-AR–independent mechanistic target of propranolol and atenolol in IH (9). Since the genetic causes for IH are mostly unknown, deciphering the molecular determinants that promote the tumor growth is of fundamental importance for the advancement of this field. The use of R(+) enantiomers or SOX18 inhibitors could increase efficacy and safety, while minimizing potential side effects. Some studies of IH proposed that propranolol can promote adipogenesis and thereby speed up the onset of the tumor regression phase. In future investigations, it will be important to determine whether SOX18 is involved in tumor involution. Additional studies could also unravel other SOX18 transcriptional targets directly involved in IH vasculogenic growth. Furthermore, as SOX18 is a major regulator of angiogenesis and lymphangiogenesis, it is exciting to speculate that it plays a major role in other vascular and/or lymphatic anomalies. Selective SOX18 inhibitors could be used in the future for the treatment of IH and related diseases.

## Version 1 02/01/2022

Electronic publication

## References

[1] Luu M, Frieden IJ (2013). Haemangioma: clinical course, complications and management. Br J Dermatol.

[2] Boscolo E, Bischoff J (2009). Vasculogenesis in infantile hemangioma. Angiogenesis.

[3] Black JW (1964). A new adrenergic betareceptor antagonist. Lancet.

[4] Mehvar R, Brocks DR (2001). Stereospecific pharmacokinetics and pharmacodynamics of beta-adrenergic blockers in humans. J Pharm Pharm Sci.

[5] Leaute-Labreze C (2008). Propranolol for severe hemangiomas of infancy. N Engl J Med.

[6] Leaute-Labreze C (2015). A randomized, controlled trial of oral propranolol in infantile hemangioma. N Engl J Med.

[7] Stiles J (2012). Propranolol treatment of infantile hemangioma endothelial cells: A molecular analysis. Exp Ther Med.

[8] Wong A (2012). Propranolol accelerates adipogenesis in hemangioma stem cells and causes apoptosis of hemangioma endothelial cells. Plast Reconstr Surg.

[9] Seebauer CT (2021). Non–beta blocker enantiomers of propranolol and atenolol inhibit vasculogenesis in infantile hemangioma. J Clin Invest.

[10] Khan ZA (2008). Multipotential stem cells recapitulate human infantile hemangioma in immunodeficient mice. J Clin Invest.

[11] Bayart CB (2017). Atenolol versus propranolol for treatment of infantile hemangiomas during the proliferative phase: a retrospective noninferiority study. Pediatr Dermatol.

[12] Lee D (2014). Propranolol targets the contractility of infantile haemangioma-derived pericytes. Br J Dermatol.

[13] Lanfranconi S (2020). Propranolol for familial cerebral cavernous malformation (Treat_CCM): study protocol for a randomized controlled pilot trial. Trials.

[14] Li W (2022). Propranolol inhibits cavernous vascular malformations by beta1 adrenergic receptor antagonism in animal models. J Clin Invest.

[15] Cermenati S (2008). Sox18 and Sox7 play redundant roles in vascular development. Blood.

[16] Herpers R (2008). Redundant roles for sox7 and sox18 in arteriovenous specification in zebrafish. Circ Res.

[17] François M (2008). Sox18 induces development of the lymphatic vasculature in mice. Nature.

[18] Young N (2006). Effect of disrupted SOX18 transcription factor function on tumor growth, vascularization, and endothelial development. J Natl Cancer Inst.

[19] Chiang IKN (2017). SoxF factors induce Notch1 expression via direct transcriptional regulation during early arterial development. Development.

[20] Hosking BM (2004). The VCAM-1 gene that encodes the vascular cell adhesion molecule is a target of the Sry-related high mobility group box gene, Sox18. J Biol Chem.

[21] Fontijn RD (2008). SOX-18 controls endothelial-specific claudin-5 gene expression and barrier function. Am J Physiol Heart Circ Physiol.

[22] Sacilotto N (2013). Analysis of Dll4 regulation reveals a combinatorial role for Sox and Notch in arterial development. Proc Natl Acad Sci U S A.

[23] Irrthum A (2003). Mutations in the transcription factor gene SOX18 underlie recessive and dominant forms of hypotrichosis-lymphedema-telangiectasia. Am J Hum Genet.

[24] Overman J (2017). Pharmacological targeting of the transcription factor SOX18 delays breast cancer in mice. Elife.

[25] Fontaine F (2017). Small-molecule inhibitors of the SOX18 transcription factor. Cell Chem Biol.

